# Something old, something new: challenges and developments in *Aspergillus niger* biotechnology

**DOI:** 10.1042/EBC20200139

**Published:** 2021-07-26

**Authors:** Timothy C. Cairns, Lars Barthel, Vera Meyer

**Affiliations:** Chair of Applied and Molecular Microbiology, Institute of Biotechnology, Technische Universität Berlin, Straße des 17. Juni 135, 10623 Berlin, Germany

**Keywords:** Aspergillus niger, citric acid, glucoamylase, macromorphology, micro-computer tomography, secondary metabolite

## Abstract

The filamentous ascomycete fungus *Aspergillus niger* is a prolific secretor of organic acids, proteins, enzymes and secondary metabolites. Throughout the last century, biotechnologists have developed *A. niger* into a multipurpose cell factory with a product portfolio worth billions of dollars each year. Recent technological advances, from genome editing to other molecular and omics tools, promise to revolutionize our understanding of *A. niger* biology, ultimately to increase efficiency of existing industrial applications or even to make entirely new products. However, various challenges to this biotechnological vision, many several decades old, still limit applications of this fungus. These include an inability to tightly control *A. niger* growth for optimal productivity, and a lack of high-throughput cultivation conditions for mutant screening. In this mini-review, we summarize the current state-of-the-art for *A. niger* biotechnology with special focus on organic acids (citric acid, malic acid, gluconic acid and itaconic acid), secreted proteins and secondary metabolites, and discuss how new technological developments can be applied to comprehensively address a variety of old and persistent challenges.

## The expanding *Aspergillus niger* product portfolio: 1917–2021 and beyond

**Figure 1:**
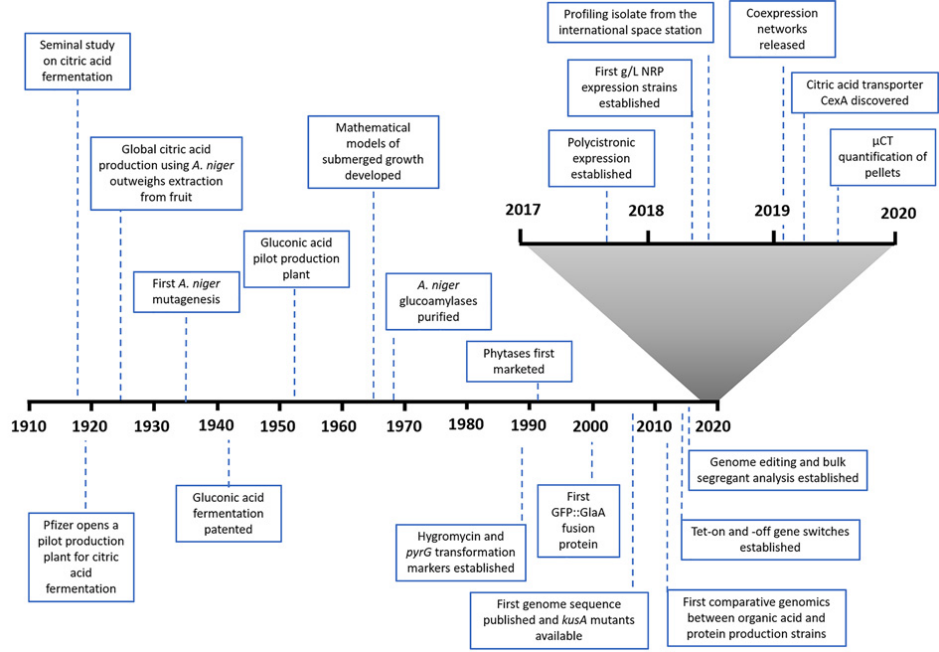
Technological and biological highlights from 100 years of *A. niger* biotechnology

For over a century, the mould *Aspergillus niger* has been gradually harnessed and modified into a multipurpose cell factory capable of converting a variety of cheap substrates into a range of valuable molecules (Figure 1 and Table 1). This global biotechnological revolution began in the 1920s, when Pfizer developed fermentation of citric acid from simple sugars using *A. niger* [1–3]. Around 20 years later, gluconic acid production using this fungus was also industrially successful [4,5]. Both these weak acids and their salts are used in a variety of applications as antioxidants, preservatives, acidulants, pH regulators or flavour enhancers in food, pharmaceutical and cosmetic industries. *A. niger* organic acid fermentation continues to grow annually, with citric and gluconic acid titres reported of 200 and 80 g/l, and markets predicted to reach 3.2 and 1 billion dollars by 2023 and 2027, respectively [6–9].

**Table 1:** Summary of key features for various *A. niger* isolates used in biotechnology

Strain	Industrial relevance	Genome accession number	Genome key findings	NHEJ mutant available	Associated companies	References
CBS 513.88	Protein (glucoamylase) producer	NCBI GCA_000002855	Seminal; over 14,000 genes predicted. Also identified putative secondary metabolite loci at genome level, RNA silencing pathways, and repeat induced point mutation	MA70.15 MA169.4	DSM (Netherlands)	[35,60,98]
ATCC1015	Citric acid producer	NCBI GCA_000230395	Gene duplication expanded genes necessary for the production of the citrate precursor oxaloacetate	No	JGI (USA)	[99]
SH2	Aconidial isolate and protein producer	No genome sequence publicly available	11,517 predicted genes; strain is aconidial likely due to lack of *prpA* gene. Strain contains multiple SNPs in cell wall biosynthetic enzyme encoding genes	No		[100]
An76	Efficient lignocellulose degrader	DNA Data Bank of Japan BCMY00000000	10,373 protein-coding genes, of which 79 are annotated to encode glycoside hydrolase	No		[101,102]
ATCC 10864	Biofilm forming isolate	NCBI MCQH00000000	10,804 predicted genes, reason for biofilm formation phenotype unclear	No		[103]
WT-D	Citric acid producer	No genome sequence publicly available		D-10	Shanghai Industrial Microbiology Institute Tech. Co (China)	[94]
LDM3	Aconidial isolate and glucoamylase producer	NCBI VTFN00000000	Non-synonymous mutations in 656 ORFs related to protein translation/ modification/secretion relative to CBS 513.88. SNP in *tupA* may cause aconidial phenotype	No	Longda Biotechnology (China)	[104]
JSC-093350089	International Space Station isolate	NCBI MSJD00000000	Modified secondary metabolite profile relative to relative to ATCC1015 due to INDELs within promoter region of developmental regulator *flbA*	CW12003	International Space Station/NASA (USA)	[26]

Note that a total of 17 genomes are available [10]. Abbreviations: NHEJ, non-homologous end-joining (used to increase targeting of exogenous DNA in recipient genome usually via *kusA* deletion or disruption, see main text); SNP: single nucleotide polymorphism; INDEL: insertion or deletion.

Other useful organic acids may soon be produced by *A. niger* at an industrial scale. Malic acid, for example, is currently used in food and pharmaceutical industries, and is a potentially sustainable replacement for the commodity chemical maleic anhydride [10]. Although malic acid is mainly produced chemically from fossil derived substrates, recent studies have engineered enhanced titres in submerged *A. niger* fermentation (e.g., 200 g/l), suggesting bioproduction of this product may soon be economically feasible [11–13]. Additionally, new acids have been added to the *A. niger* product portfolio, as evidenced by engineering itaconic acid producing isolates by heterologous expression of *A. terreus* biosynthetic and transport genes [14].

The other major application of *A. niger* is enzyme production, which broadly began in the late 1950s when advances in chromatography purification technology enabled biotechnologists to study a diverse repertoire of useful proteins. This included lactoferrin, lipase, arabinase, asparaginase, catalase, cellulase, β-galactosidase, glucoamylase, pectinase, phytase, proteases, xylanase, glucose oxidase and hemicellulase, amongst others [15]. The most successful application of these enzymes is probably glucoamylase saccharification of starch to glucose, a technology used by several multinational biotechnological companies worth over a billion dollars per year [16]. Fermentation of the glucoamylase enzyme GlaA in *A. niger* reaches titres of 30 g/l [17].

In the last decades, *A. niger* has also emerged as a producer of secondary metabolites (SMs) with titres of several g/l [17,18]. For example, heterologous expression of a *Fusarium oxysporum* non-ribosomal peptide synthetase (NRPS) encoding gene produced g/l titres of enniatin [19], which was previously developed into the antimicrobial drug fusafungine [20]. Excitingly, by modifying growth media amino acids, new-to-nature enniatin variants could be produced [19]. Additionally, by domain swap experiments in the NRPS encoding gene, production of hybrid non-ribosomal peptides which showed enhanced antimicrobial activity was possible [21].

Most recently, a new frontier in *A. niger* biotechnology has emerged, as applications of this fungus have looked to earth’s orbit and beyond. *A. niger* is one of the few fungi known to grow robustly inside the international space station [22–25], and metabolite profiling suggests astronautic isolates generate elevated levels of the antioxidant pyranonigrin A, which may enhance protection to radiation [26]. It is therefore hypothesized that *A. niger* is an outstanding candidate for use in a variety of space biotechnological applications, from generating useful products during extended space flight to terraforming efforts [25].

## Biosynthesis of proteins, acids or secondary metabolites

A huge investment of *A. niger* research has determined precise abiotic growth conditions necessary for optimal product titres, ranging from pH, carbon source, spore inoculum density, oxygenation, among many other parameters (for a recent review, see [27]). More recently, a combination of molecular, cellular and omics approaches has been used to comprehensively understand the molecular and cellular mechanisms of product biosynthesis and secretion/export.

### Organic acid synthesis and export

Most industrially relevant organic acids produced by *A. niger* are metabolites of the tricarboxylic acid cycle (TCA), which are produced under specific growth stages and in response to various abiotic cues (e.g., phosphate limitation, low pH [28,29]). One notable acid that is broadly independent from the TCA cycle is gluconic acid, which is biosynthesized from a glucose substrate by the extracellular, cell wall-localized glucose oxidase GoxC [30].

Citric acid is formed from precursors oxaloacetate, acetyl-CoA and water at the final stage of the TCA by the mitochondrial citric acid synthase CitA [31]. Citric acid that is not recycled back into the TCA cycle can be transported to the cytosol by malate/citrate shuttles in the mitochondrial membrane including CtpA [32], which can then be exported from the cell by the recently discovered putative major facilitator superfamily transporter CexA [33]. Precisely why *A. niger* secretes citric acid into the external media is unknown, but this may occur after a diauxic shift to phosphate limited growth [28], and to increase iron bioavailability [29]. Genetic engineering of genes encoding TCA enzymes and substrate transporters have reported varied success in citric acid biotechnological applications. For example, over-expression of *citA* [31] and other TCA pathway enzymes [34] does not increase titres of citric acid, and disruption of *ctpA* does not impact productivity [32]. In contrast, over-expression of the CexA transporter encoding genes using either the inducible Tet-on system [35,36] or high constitutive expression increases citric acid titres by approximately 5 and 3 times, respectively [33].

Fermentation of the emerging value product malic acid is highly interconnected with that of citric acid due to the role of malic acid the TCA cycle. Additionally, as noted above, cytosolic malic acid is thought to be imported into the mitochondria by the malate/citrate shuttle CtpA. Increase of cytosolic malic acid has therefore been hypothesized to initiate citric acid fermentation in *A. niger* due to the exchange of malic acid and citric acid across the mitochondrial membrane [37]. However, it should be noted that *ctpA* mutants display comparable levels of both citric and malic acid in culture filtrates compared with progenitor controls – possibly due to functional redundancy of this transporter – thus limiting applications for genetic engineering of CtpA [32].

Despite these challenges, malic acid titres can be successfully increased by genetic engineering of *A. niger* membrane transporters, in a comparable approach to that of CexA and citric acid. The major *A. niger* malic acid exporter was identified by homology searches using the sequence from the fission yeast malate/succinate proton symporter Mae1 [12]. By overexpression of the *A. niger* orthologous gene, named *dctA*, malic acid titres could be increased by over 20% compared with controls [12]. Elsewhere, it was recently demonstrated that engineering *cexA* can be used to hyperproduce malic acid [11]. Disruption of *cexA* concomitantly reduces titres of citric and malic acid, with the mutant having reduced expression of genes for glucose transportation and the glycolytic pathway [11]. By overexpressing key genes in the *cexA* disruption mutant, including a low affinity glucose membrane transporter *mstC*, hexokinase *hxkA* and pyruvate kinase *pkiA*, among other genes, malic acid titres could be increased by ∼23% compared with the progenitor control [11]. Taken together, recent functional characterizations of CexA and other membrane transporters can be viewed as breakthroughs in *A. niger* biotechnology, which may soon be applied to generate organic acid hyper-producing isolates at an industrial level, especially when combined with other recent genetic strategies for maximizing productivity [13,38]. More generally, discovery and characterization of all *A. niger* organic acid organelle and plasma membrane transporters, and their detailed mechanistic functions, are crucial future objective for biotechnologists.

It is also notable that *A. niger* is a potential chassis host for production of itaconic acid, an emerging bulk chemical commercially produced by *A. terreus* [39]. In this species, cis-aconitate from the TCA cycle is transported out of mitochondria by the transporter MttA, after which it is converted to itaconate by the *cis-*aconitate decarboxylase CadA [14]. Ultimately, itaconate is exported from *A. terreus* by the membrane transporter *mfsA* [14]. *A. niger* lacks a known *cadA* orthologue, and expression of a codon optimized *cadA* and *mttA* was sufficient for proof of principle production of itaconic acid, with export from the cell presumably performed by a native membrane transporter [14]. By overexpressing the *A. niger* cytosolic citric acid synthase *citB* [40], and by identification and deletion of itaconic acid bioconversion pathways [41], titres above 30 g/l could be achieved in subsequent studies, which may enable the use of this molecule as a sustainably produced bulk chemical.

### Protein secretion

The classical protein secretion route involves trafficking of cargo-loaded vesicles along microtubules and actin cables from the endoplasmic reticulum (ER)/Golgi to the hyphal tip [42–44]. Vesicles cluster at a sub-apical site called the Spitzenkörper, and become fused with the plasma membrane by a multiprotein complex termed the exocyst [45], after which cargo is released. In addition to extracellular enzymes for nutrient hydrolysis, cell wall synthesizing proteins are trafficked in vesicles, hence coupling growth and secretion at the hyphal tip. Exocytic addition of vesicle membrane to the plasma membrane is balanced by endocytosis, most likely at a sub-apical actin ring, which is important to maintain hyphal polarity [46]. The majority of industrially important enzymes probably follow this route, as evidenced by N-terminal secretion signals, and several studies that localize enzymes such as the glucoamylase GlaA to secretory vesicles (46–48). Modulation of classical secretion, for example by titratable expression of GTPase regulators of vesicle trafficking [46], or by constructing mutants with branching defects and elevated hyphal tip numbers [47,49] is an effective method for elevating secretion of both total protein and enzymes such as GlaA.

In addition to vesicle trafficking to the hyphal tip, other non-classical routes certainly play a role in *A. niger* protein secretion. There is growing evidence that proteins may exit hyphae at intercalary regions, most notably septal junctions [46,48,50–52], which could conceivably be an extracellular route for industrially relevant proteins, including GlaA [46]. Additionally, several proteins that lack any secretion signal for translocation to the ER are found in culture supernatants [53]. The *A. niger* aspartic protease PepN is secreted by non-classical routes, which are independent of autophagic processes necessary for unconventional secretion in *Saccharomyces cerevisiae* [54]. Excitingly, extracellular vesicles from a member of the fungal kingdom were first characterized in *Cryptococcus neoformans* [55] despite their probable observation in *A. nidulans* 40 years earlier [56]. Extracellular vesicles thus might exist in *A. niger.* In other fungi they can contain proteins, nucleic acids, carbohydrates, and lipids (reviewed in [57]), and could thus be relevant for industrial applications. Currently, mechanistic understanding of these unconventional secretion pathways is limited, and discoveries in this area may enable new avenues for elevating titres of proteins and other products during fermentation.

### Secondary metabolites

Most *A. niger* SMs are produced during low or zero growth [58] by biosynthetic gene clusters (BGCs) consisting of one or more key enzyme encoding genes, such as a polyketide synthase (PKS) or NRPS [59]. These enzymes are modular, and function respectively by loading acetyl-CoA or an amino acid substrate onto a carrier protein. Next, the molecule is elongated and chemically modified by various modules, after which a nascent SM is released from the carrier protein through activity of a thioesterase domain [59]. Enzymes normally encoded by contiguous genes within the BGC then modify of the core moiety, for example by glycosylation, acylation or methylation. The BGC may also encode membrane transporters necessary for internal transport/export of the SM, and transcription factors which regulate BGC expression.

The first *A. niger* genome sequence was published in 2007 [60], with 78 predicted BGCs putatively involved in SM biosynthesis [61], the highest in any Aspergillus species. The majority of these BGCs produce an unknown product; recent data mining from 283 microarray experiments under a diverse range of experimental conditions suggests that most are either transcriptionally silent in the lab, or expressed in strictly limited circumstances [62]. Consequently, the potential for experimentally activating *A. niger* BGCs by specialized (co)cultivation conditions are promising for new bioactive molecule discovery [63]. Molecular approaches for SM activation have also been used in other filamentous fungi, including overexpression of the NRPS/PKS/BGC transcription factor, or through modification of epigenetic regulators which modulate BCG expression by chromatin remodelling [64].

Biosynthesis of most SMs is speculated to be highly compartmentalized and spatially organized in the cell. For example, penicillin synthesis in *Penicillium chrysogenum* (which can be considered one of the best characterized SM biosynthetic pathways) includes storage of amino acid precursors in vacuoles, biosynthesis both in the cytosol and peroxisomes, and secretion by either a hypothetical membrane transporter or in secretory vesicles (for a recent review see [65]). In *A. nidulans*, which also produces penicillin, targeting of the cytosolic NRPS AcvA to peroxisomes significantly increased titres [66], highlighting how understanding and modulating biosynthetic enzyme subcellular localization can be an effective future strategy for *A. niger*.

### Connecting three product groups

Traditionally, *A. niger* biotechnological research has been separated into protein, organic acid and SM sub-disciplines. There is however a recent trend to holistically study these related fields and to understand primary metabolism including redox and cofactor metabolism as foundational basis for all products formed [1,3,67]. One recent discovery that has connected all three disciplines began by investigating a non-acidification phenotype in the UV mutagenized *A. niger* isolate D15 [68]. Non-acidification of growth media is of major interest for enzyme production isolates, as secretion of acid-induced proteases will be inhibited [69].

Accordingly, bulk segregant analysis was applied to mutant D15, a powerful technique which is broadly applicable to identify single-nucleotide polymorphisms (SNPs) in many mutagenized *A. niger* strains (Figure 1, [68,70]). In this approach, parasexual crosses are induced on solid medium between the strain of interest and a non-mutagenized control by selection and subsequent counter selection of heterokaryotic mycelia. Segregant progeny displaying the phenotype of interest – such as non-acidification of growth media – then have DNA pooled and bulk sequenced at sufficient depth so that multiple SNPs can be identified [70]. Ultimately, the SNP that causes the phenotype of interest is conserved in all the progeny, and absent in the control isolate.

Intriguingly, this approach demonstrated that non-acidification by *A. niger* mutant D15 was due to a SNP in the putative methyltransferase gene encoding a master regulator of secondary metabolism LaeA [68]. A follow up study demonstrated that LaeA regulates acidification by histone H3K4 and H3K9 methylation in the *cexA* promoter of the shochu producer *A. luchuensis* mut. *kawachii* [71], thus likely mechanistically explaining the non-acidification of *A. niger* D15 [68]. From SM perspectives, deletion and overexpression approaches have confirmed the role of *A. niger* LaeA in regulating hundreds of *A. niger* SM genes [72]. Finally, another most recent study linked for the first time the intracellular redox status of *A. niger*, which is dependent from the sugar and organic acid metabolism, with protein and glucoamylase production. It was demonstrated that metabolic engineering through NADPH cofactor engineering can indeed cause a substantial increase in GlaA titers [67].

Many more insights into the interconnections between organic acid, protein and secondary metabolite production are likely to be revealed in future studies, which may enable rational design of hyperproduction strains. Despite the above advances, several challenges exist for (re)engineering *A. niger* as a maximally efficient cell factory. Two key challenges are discussed below.

## How can we measure and control filamentous growth and macromorphology?

The control of filamentous morphology in an industrial *A. niger* application is crucial for any kind of submerged production process and can happen on two basic levels, micromorphology and macromorphology, describing the structure of individual hyphae and the characteristics of an entire aggregate of mycelium, respectively (reviewed in [27]). Whereas the beneficial impact of altering micromorphology of *A. niger* has been established in some instances (e.g., a hyperbranching phenotype on protein productivity [27]), a significant challenge is precise, user defined marcomorphological measurement and control in submerged culture. Macromorphology of *A. niger* in submerged culture can be divided into three classes: dispersed mycelium (free, individual hyphae), clumps (groups of hyphae loosely attached, forming undefined aggregates) and pellets (dense, spherically symmetric hyphal aggregates with a spore centre, originating from co-aggulative pellet forming mechanism) [73,74]. While dispersed cultures show a higher viscosity, thus lower mixability and oxygen transfer in the culture broth, the nutrient supply stays uniformly stable throughout the entire population. In a pellet forming process on the other hand, the mixability and oxygen transfer in the culture broth are higher, but the densely packed hyphal structures provoke oxygen and nutrient gradients within single pellets, thus causing limitations inside the pellet core [27]. Due to the often described, yet not fully understood correlation between growth/morphology and productivity in *A. niger*, both morphological forms can be favourable, depending on the desired product [75,76]. As classical, vesicle-based protein secretion and growth are strictly coupled processes, the well nutrient-supplied, thus fast-growing dispersed hyphae as well as the outer, non-nutrient limited regions of pellets are assumed to be ideal production hosts. For the production of secondary metabolites, which peaks at zero or very low growth, the inactive cores of pellets are probably well suited [77].

The predominant macromorphology present in a culture is classically controlled by process parameters like inoculum, medium composition, pH, temperature or mechanical forces [78]. In addition to this, novel approaches in the field of genetic engineering allow the titration of macromorphology without changing process conditions. It was for example shown recently, that the reduced expression of the putative gamma-adaptin encoding gene (*aplD*), results in a hyperbranching phenotype with defects in pellet formation, and could thus give a possible target for morphology engineering [75].

While dispersed mycelia can be analysed comparatively easily using light microscopy, this technique is not sufficient to image the complex, 3D structure of filamentous pellets, and especially their cores. To overcome this problem, pellet-slicing and subsequent light microscopic investigation has become a common tool [79]. The obvious disadvantage of this procedure is the destructive nature of slicing. Other approaches to examine pellets include confocal laser scanning microscopy of previously frozen and died slices, allowing to gain 3D insights into parts of the pellet's structure [80] as well as scanning electron microscopy of entire pellets, unveiling the difference between highly intertwined superficial hyphae and a densely packed mycelium [81]. In addition to these imaging-based methods, flow cytometry can be used to get information about the core compactness of fungal pellets [82]. However, none of these methods allow a non-destructive, detailed morphological analysis of an entire pellet. This problem was recently addressed with X-ray microtomography (µCT) [83]. Here, entire freeze-dried fungal pellets were investigated using X-ray and computer-based image analysis techniques, giving the exact distribution of hyphae and the number of hyphal tips and branches inside a pellet. The key advantage of this novel µCT technique is thus the possibility to determine the exact inner structure of intact fungal pellets and to calculate the effective diffusion factor inside an *A. niger* pellet in relation to the hyphal fraction [84]. Remarkably, it was shown that the correlation between effective diffusion factor and hyphal fraction in fungal pellets follows a universal law, which is independent of micromorphological features like hyphal diameter, branching angle or interval and growth angle [85]. With this new tool, it is conceivable to calculate the actual nutrient or oxygen gradient within a pellet in a foreseeable future. To do so, two major elements must be developed; a term describing the uptake or production rate inside the pellet and a second term for the diffusive mass transport through the pellet. As soon as both these elements come together, a detailed distribution of each individual substance inside fungal pellets can be predicted.

**Figure 2:**
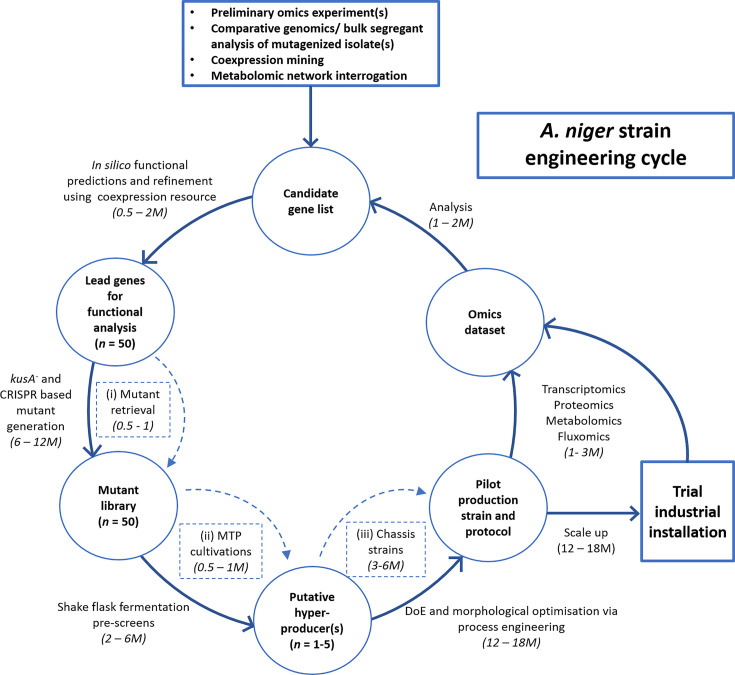
Current and future *A. niger* strain engineering cycles The cycle begins with a candidate gene list(s) for functional analysis derived from previous datasets/experiments. Numbers in parentheses denote either the total amount of time for each step (months per scientist, M) or an exemplar number of genes remaining in each step (e.g., *n* = 50 candidate genes). Refinement of functional predictions is now possible by publicly available coexpression resources for *A. niger*. Mutants can be generated in-house using *kusA* mutants/genome editing technology, or in future from acquiring isolates from a mutant library resource (dotted arrow). Next, preliminary fermentation can be conducted, currently in shake flask, but in future via MTP cultivation. Putative hyperproducers (which in this example has generated 1-5 isolates) can then be tested via extensive design of experiment (DoE) approaches or, in future, by functional analysis in pre-generated chassis strains with a defined macromorphology. Pilot production strains can then be further analysed for another iteration of strain engineering, or taken for scale up during trial industrial production. This engineering cycle is similar for other industrially used filamentous fungi, both with regards to the main steps, time-frames, and major bottle-necks.

By combining the techniques described here, hopefully it will shortly be possible to design optimized macromorphology in each individual *A. niger* process as follows: (i) depending on the desired product, a suitable overall macromorphology is selected (dispersed/pellet). If the chosen form is pelleted, optimization is needed: (ii) nutrient gradient calculation to reverse engineer a theoretical pellet structure, optimized in nutrient and oxygen supply for the production of the specific substance; (iii) genetic and process engineering techniques to adjust the macromorphology to the calculated optimum; (iv) X-ray microtomography or other methods to examine the resulting macromorphological structures. Consequently, morphology engineering of *A. niger* chassis isolates with a pre-programmed macromorphology in submerged culture will drastically reduce screening time for putative hyperproduction mutants (Figure 2).

## Are high-throughput assays of submerged culture possible?

Two key technological developments have drastically increased the throughput of *A. niger* null, overexpression and conditional-expression mutant construction. Firstly, deletion of non-homologous end joining components significantly elevate the rates of homologous recombination between exogenous DNA cassettes and the *A. niger* genome, thus increasing targeting of desired sequences at user define loci [86]. In *A. niger*, this is conventionally achieved by deletion or disruption of the *kusA* gene, which encodes a subunit of the Ku heterodimer [87]. Such *kusA* mutants are available in organic acid and protein industrial strain lineages (Table 1). Secondly, genome editing using Cas9, Cas12a and other guided nucleases now enable rapid and precise deletion, disruption and insertion of desired sequences throughout the genome [88–90]. Consequently, it is now conceivable for single studies to generate libraries of several dozen or even hundreds of mutants with putatively elevated product titres.

Despite these advances, a significant bottleneck to screening such *A. niger* libraries for improved productivity is the limited throughput of shake flask and bioreactor based submerged culture, which are limited to several dozen technical replicates per user/week (Figure 2). Microtiter assays of liquid growth have been widely used in instances where macromorphological development is broadly irrelevant, for example high-throughput measurements of antifungal efficacy against *A. niger* [91]. However, it is currently unclear if it is possible to meaningfully replicate macromorphological changes and productivity observed in bioreactors (L) or shake flasks (ml) in smaller volumes (µl). Encouragingly, there has been success in culture miniaturization using other filamentous fungal cell factories. For example, a microtitre plate assay for screening dozens of *A. vadensis* transformants expressing an *A. niger* feruloyl esterase B, an enzyme required for dissociation of lignin from hemicellulose was developed [92]. High-throughput screens of submerged growth enabled clones with undetectable or minimal levels of overexpressed protein to be rapidly identified. Importantly, this study demonstrated that enzyme activity of clones was qualitatively comparable at either shake flask or microtiter scales. However, the authors observed an absolute decrease in enzyme activity around tenfold between shake flask and microtiter cultivations [92]. Therefore, despite the obvious advantages in throughput using this approach, microtiter cultivation may not be appropriate where detection of the desired product is challenging. Others were able to reproducibly ferment approximately 80g/l itaconic acid using *A. terreus* bioreactor (400 ml), shake flask (100 ml) and 96 well (100 µl) cultivations [93]. As the authors noted, the small size of *A. terreus* pellets (∼100 µm diameter) probably enabled comparable productivity across cultivation platforms. Are such scale down methods possible for *A. niger*, where pellets in shake flask/bioreactor models of either protein or citric acid fermentation reach over several mm in diameter [75,94]?

An innovative approach combines microparticle based reduction in *A. niger* pellet size with growth in microtiter wells [95]. By modifying inoculum and microparticle density, it is possible to both elevate production of a food flavouring compound in this system, while reproducibly generating pellets of uniform size [95]. In another study, microtiter cultivation of *A. giganteus* was combined with online measurements of fungal growth using scattered light in the BioLector system [96]. This approach also relied on reducing pellet formation, specifically by supplementing growth media with CaCl_2_ [96]. This approach was further developed into an automated workflow for BioLector-assisted microtiter plate cultivation of *A. carbonarius* that included an automated pipeline of sample processing, injection, image acquisition and morphology analysis [97]. These studies therefore demonstrate that reproducible growth in microtiter format of pellet forming fungi is indeed possible; however this currently requires modification of culture conditions to inhibit formation of large pellets. Therefore, while biotechnologists can currently apply high-throughput microtiter cultivations in *A. niger*, a limitation to this approach is that certain products which are favourably or exclusively secreted by large pellets will not be detectable [27]. Addressing this bottleneck will drastically increase rational strain engineering throughput in the future (Figure 2).

## Summary

- *A. niger* is a microbial cell factory used to produce proteins, organic acids, and secondary metabolites.
- The molecular, cellular and metabolic basis of product biosynthesis and secretion are currently being revealed, suggesting they are highly interconnected.
- The existing *A. niger* toolkit and available resources promise a rapid period of genetic, metabolic, and morphology engineering for efficient cell factories.

## Abbreviations

BGC: biosynthetic gene cluster
INDEL: insertion or deletion
NHEJ: non-homologous end-joining
NRPS: non-ribosomal peptide synthetase
PKS: polyketide synthase
SM: secondary metabolite
SNP: single nucleotide polymorphism
